# An Integrative Review of Computational Methods Applied to Biomarkers, Psychological Metrics, and Behavioral Signals for Early Cancer Risk Detection

**DOI:** 10.3390/bioengineering12111259

**Published:** 2025-11-17

**Authors:** Lucia Bubulac, Tudor Georgescu, Mirela Zivari, Dana-Maria Popescu-Spineni, Cristina-Crenguţa Albu, Adrian Bobu, Sebastian Tiberiu Nemeth, Claudia-Florina Bogdan-Andreescu, Adriana Gurghean, Alin Adrian Alecu

**Affiliations:** 1 Department of Family Medicine, Faculty of Medicine, “Carol Davila” University of Medicine and Pharmacy, 020021 Bucharest, Romania; lucia.bubulac@umfcd.ro; 2 Faculty of Medicine and Pharmacy, University of Oradea, 410073 Oradea, Romania; georgescu.tudor@student.uoradea.ro; 3 Department of Psychology and Educational Sciences, Faculty of Psychology, Ecological University of Bucharest, 061341 Bucharest, Romania; mirela.zivari@ueb.education; 4 Department of Psychology, Emergency University Hospital, 050098 Bucharest, Romania; 5 Department of Social Medicine, Faculty of Midwifery and Nursing, “Carol Davila” University of Medicine and Pharmacy, 020021 Bucharest, Romania; spineni.popescu@umfcd.ro; 6 Department of Biomedical Anthropology, “Francisc Reiner” Institute of Anthropology of the Romanian Academy, 050711 Bucharest, Romania; 7 Department of Genetics, Faculty of Dentistry, “Carol Davila” University of Medicine and Pharmacy, 020021 Bucharest, Romania; 8 Faculty of Automatic Control and Computers, National University of Science and Technology Politehnica, 060042 Bucharest, Romania; 9 Pharmacognosy Discipline, Faculty of Medicine and Pharmacy, University of Oradea, 410028 Oradea, Romania; snemeth@uoradea.ro; 10 Department of Speciality Disciplines, “Titu Maiorescu” University, 031593 Bucharest, Romania; claudia.andreescu@prof.utm.ro; 11 Department of Internal Medicine, Faculty of Medicine, “Carol Davila” University of Medicine and Pharmacy, 020021 Bucharest, Romania; adriana.gurghean@umfcd.ro; 12 Faculty of Engineering in Foreign Languages, National University of Science and Technology Politehnica 060042 Bucharest, Romania; alin_adrian.alecu@upb.ro

**Keywords:** artificial intelligence, early cancer risk, digital screening, biomarkers, psychology, behavior, multimodal data fusion, convergence concept, structured informational management

## Abstract

The global rise in cancer incidence and mortality represents a major challenge for modern healthcare. Although current screening programs rely mainly on histological or immunological biomarkers, cancer is a multifactorial disease in which biological, psychological, and behavioural determinants interact. Psychological dimensions such as stress, anxiety, and depression may influence vulnerability and disease evolution through neuro-endocrine, immune, and behavioural pathways, especially by affecting adherence to therapeutic recommendations. However, these dimensions remain underexplored in current screening workflows. This review synthesizes current evidence on the integration of biological markers (tumor and inflammatory biomarkers), psychometric profiling (stress, depression, anxiety, personality traits), and behavioural digital phenotyping (facial micro-expressions, vocal tone, gait/posture metrics) for potential early cancer risk evaluation. We examine recent advances in computational sciences and artificial intelligence that could enable multimodal signal harmonization, structured representation, and hybrid data fusion models. We discuss how structured computational information management may improve interpretability and may support future AI-assisted screening paradigms. Finally, we highlight the relevance of digital health infrastructure and telemedical platforms in strengthening accessibility, continuity of monitoring, and population-level screening coverage. Further empirical research is required to determine the true predictive contribution of psychological and behavioural modalities beyond established biological markers.

## 1. Introduction

Cancer remains a major global public health challenge, ranking as the second leading cause of morbidity and mortality worldwide after cardiovascular diseases. Projections indicate that by 2050, cancer-related annual deaths will reach 18.5 million, representing an 89% increase compared to 2020. In 1990, there were 8.1 million new cancer cases and 5.2 million cancer-related deaths worldwide. By 2022, these figures had grown to 19.97 million new cases and 9.75 million deaths, with the five-year prevalence reaching 53.5 million cases. In Europe, the cancer incidence increased by 58% between 1995 and 2022, rising from 2.05 to 3.24 million cases. This trend is also evident at the national level: according to GLOBOCAN 2018 [1], Romania reported 83,461 new cancer cases in 2018. By 2022, this number had risen to 104,000, with 50,902 cancer-related deaths, placing Romania ninth in Europe for cancer mortality [2,3].

### 1.1. Motivation for Early AI-Based Cancer Detection

Early cancer detection dramatically improves survival, but current clinical pathways still fail to detect disease before symptoms become clinically visible. Biological signals appear much earlier than classical clinical thresholds, yet they are weak, fragmented across biological, behavioural, and digital domains, and impossible to interpret by human clinicians alone. In this context, AI can integrate heterogeneous signals, detect emerging patterns before clinical manifestation, and enable a transition from late reactive diagnosis to early proactive screening.

### 1.2. Conventional Screening Limitations

Current conventional screening systems remain costly, invasive, infrequent and uni-modal, often relying on isolated biomarkers or single imaging events. These constraints, combined with a limited frequency of testing, poor scalability and unequal access, lead to systematic delays in detection and under-diagnosis in early stages. Furthermore, subtle phenotypic signals (e.g., micro-behavioural shifts) are systematically ignored because they do not fit into the classical diagnostic workflow.

### 1.3. Advantages of AI and Multimodal Data Integration

AI models are inherently capable of detecting complex nonlinear patterns across highly heterogeneous sources such as voice, facial dynamics, gait, psychometric markers and high-resolution digital biomarkers. Multimodal integration enhances the signal-to-noise ratio, increases robustness, enables redundancy between modalities, and drastically increases sensitivity in early detection windows where each single signal is individually weak.

### 1.4. Novelty of This Review Compared to Existing Surveys

Previous reviews have focused on single modalities (genomics, radiomics, blood biomarkers, or wearable signals) or have analyzed AI models separately. This article is the first to describe an integrated, convergent, structured AI architecture that harmonizes all these modalities into a single multimodal representation standard aimed specifically at ultra-early cancer screening. Specifically, this review uniquely synthesizes the computational methodologies that could potentially enable a future multimodal cancer vulnerability screening paradigm integrating biological, psychological, and behavioural signals.

### 1.5. Roadmap of the Manuscript

Section 2 reviews the theoretical and biological basis of oncogenesis and psychological factors, including current modalities and their AI representation models. Section 3 presents the conclusions and future directions.

### 1.6. Biological and Psychosocial Context of Cancer Vulnerability

The etiology of neoplastic disease is multifactorial, and while it is difficult to precisely quantify the contribution of endogenous and exogenous factors to oncogenesis, both categories play significant roles. Modifiable risk factors such as smoking, obesity, sedentary behavior, pollution, and exposure to heavy metals coexist with non-modifiable factors, including genetics, hormones, immune system status, age, gender, and ethnicity [4,5,6].

Besides these, psycho-emotional factors such as stress, anxiety, and depression can weaken the immune function and represent a critical, though often overlooked, dimension of cancer vulnerability [7,8]. The modern lifestyle, combined with global events such as the COVID-19 pandemic, war, and economic crises, has created conditions conducive to a rising burden of mental health disorders. According to the WHO, approximately 970 million people worldwide—one in eight individuals—were affected by a mental disorder by 2024. If in 1990, 3.5% of the adult population suffered from major depression, by 2024, this figure had risen to 5%, with an additional 4% affected by anxiety disorders. These numbers represent a 25% increase following the COVID-19 pandemic, as highlighted by recent WHO and Lancet reports [4,5]. This ascendent curve of mental disorders post-COVID-19 is synchronized with an increase in cancer incidence, over the same period of time, of 3.5% [9,10,11,12], which supports the premise that a direct relation between mental health, clinically determined by psychological and behavioral testing, and oncogenesis is probable, but not yet objectively established [13,14].

Over the last decades, for preventive purposes, multiple screening programs have been implemented to detect the early presence of histological or immunological markers that indicate the onset of neoplasia. These include PSA testing, fecal occult blood tests, pap smears, HPV testing, colposcopy, breast MRI, ultrasound, and others. However, the persistent increase in cancer incidence over the past decades highlights the limitations of current screening methods. This raises the question of what hinders the success of current preventive approaches and of what additional factors may be contributing to this rise in vulnerability, beyond the already assessed ones, mainly the environmental exposures.

In our review, we focus on two aspects that are probable causes of current methods’ drawbacks: (1) the uneven informational management, both conventional and computerized [15,16,17] in health, and hence in cancer management, and (2) the overlooking of relevant direct oncogenic factors (or potential factors), as well as of indirect, systemic factors, such as the health systems’ function and the social behavior related to health.

To date, all modern health systems’ activities are computerized, to a various degree of integration and using various technical solutions. This means that all cancer prevention, screening, and therapeutic activities, whether coordinated into programs or clinically conducted, use a type of ‘computerized informational management’, but not a unified or widely integrated one. Furthermore, medical information is formalized and structured very differently in local and national electronic health records (EHRs) or other formats, such as health insurance records, clinical studies, or pharmacovigilance reports [18,19,20,21,22]. So, there is a technological and an informational inconsistency in global health computerized data management [23,24,25]. For this major issue, a possible solution is reviewing the rational use of AI-type applications and of other integrative solutions, such as the ‘convergent computerized structured information management’.

Current artificial intelligence (AI) developments are particularly relevant for our review, since they are enabling the fusion of heterogeneous datasets into predictive models that support personalized medicine and early detection strategies. Recent developments in digital phenotyping, including facial micro-expression analysis, vocal tone profiling, and gait/posture recognition, combined with psychometric evaluations and biomarker assays, illustrate the potential of integrative multimodal screening.

Regarding the expansion of the range of certified and potential oncogenic direct factors, stress emerges as a possible incriminated element. Its effects on the human body are manifested at both psychological and physiological levels, fostering the development of anxiety disorders and depression while also promoting systemic conditions such as cardiovascular, endocrine, digestive, autoimmune, and oncological disorders. Precisely and consistently determining the level of personal stress, through objectifiable psychological testing and aggregating the results within standard EHRs, including all other clinical and paraclinical determinations currently used, could contribute to enhancing the success rate of overall cancer management, towards a holistic approach, which is considered increasingly valuable in modern oncology [26,27,28], one that integrates biological, psychological, and behavioral determinants of health.

One important determinant factor of any medical program’s success remains the social one [29,30,31,32]. This is the individual and general relation with the health system, based on education and accessibility. This relation impacts the adherence to health programs, such as to oncological preventive and therapeutic ones, patients’ compliance and resilience, with regard to prescribed preventive and therapeutical plans, and patient–professional cooperation, which is essential for medical research. Improving the social adherence to health is a key factor for cancer management’s success.

Health systems are globally hierarchized, from primary assistance to excellent health centers. Equally, health professionals’ access to paraclinical investigations and information technology is uneven. So, it is of essence to augment the clinical cancer management capabilities at all health systems’ levels, but especially in primary assistance, including pharmacies. In this area, AI-type applications, integrated into convergent platforms, could prove their potential, especially since they offer the capability of efficiently interconnecting professionals to patients, in stable telemedical networks, and healthcare providers of all levels to primary assistance, aiming towards a collaborative, ‘shared resources’ type of platform.

This study synthesizes current knowledge at the intersection of oncology, psychology, and digital health. First, it highlights the biological and psychological theoretical grounds of the stress–depression–cancer connection. Second, it examines advances in modern computational methods, such as conventional database management and AI-assisted multimodal data management, Third, it explores their current use in medical research and practice, related to cancer–psychology interdependence. Finally, it proposes and theoretically models an integration of the two, within convergent platforms, using structured information. Hence, this paper considering the opportunities and challenges for the future implementation of integrative computerized platforms in early cancer risk detection and overall cancer management.

## 2. Theoretical and Biological Basis of Oncogenesis and Psychological Factors

The connections between the nervous, endocrine, and immune systems provide crucial insights into the pathophysiological mechanisms of oncogenesis, underscoring the need for a comprehensive and multidimensional screening approach. Recent perspectives emphasize the value of integrative strategies that combine multiple complementary components—biological tumor markers, inflammatory mediators, and psychological profiling—to assess vulnerability to stress, and digital analyses such as micro-facial expression recognition, voice pattern assessment, and gait or posture evaluation. Each of these domains is supported by accumulating scientific evidence, highlighting their relevance to oncogenesis and justifying their integration into holistic cancer risk assessment frameworks.

It is also important to emphasize that, in a holistic approach, determining the exact clinical relevance of each determination category is of essence. If, for tumor markers, the relevance is high, for the inflammatory ones, it is debatable, while for the psychological determinations, it is still unclear if a rigorous assessment is further required.

Furthermore, especially for cancer early diagnosis, as well as for overall cancer management, clinical correlation of all types of determinations reviewed in this paper is absolutely necessary, while extensive studies are to be conducted in order to establish, in a measurable manner (parametrizable), the relevance of each determination, especially of the non-biological ones, as previously discussed. In this direction, the computerized methods provide indispensable support, both for data management and complex data processing, while taking into account the limitations of currently used IT systems and applications in health, as previously presented.

### 2.1. Biological Significance of Tumor Markers

Oncogenesis research has identified mechanisms that enable malignant cells to proliferate and spread, fostering chronic inflammation and promoting genomic instability through mutagenic processes such as reactive oxygen species (ROS) production [33,34,35]. Oncogenetics contributes to this field by identifying individuals with hereditary or monogenic predispositions, thereby facilitating early diagnosis, monitoring, and personalized preventive interventions. For example, a positive family history of colorectal cancer increases the risk by 2.6-fold in women and 3-fold in men [36].

Cancer cells may trigger immune responses through tumor-associated antigens (TAAs) and tumor-specific antigens (TSAs), which are relevant for screening various cancers and monitoring therapeutic responses. Examples include CA-19-9, CA-125, and CA-15-3, while oncofetal antigens such as alpha-fetoprotein (AFP) and carcinoembryonic antigen (CEA) may reappear in neoplastic cells [37]. Although clinically useful, these markers are insufficient as standalone screening tools; their value increases when integrated into broader multimodal risk assessment strategies.

Genetic susceptibility further illustrates the complexity of cancer development. Inherited mutations increase the predisposition to malignancy rather than directly transmitting the disease itself. Notable examples include mutations in MLH1 (colorectal, gastric, and endometrial cancers), BRCA1 and BRCA2 (ovarian, breast, and prostate cancers), RB1 (retinoblastoma and osteosarcoma), MEN1 (parathyroid, pancreatic, and pituitary tumors), and RET mutations associated with Multiple Endocrine Neoplasia type 2A (thyroid cancer and pheochromocytoma) [38]. Importantly, the lifestyle, mindset, perception of stress, and environmental exposures can modulate the penetrance and clinical expression of these genetic alterations [39].

Beyond classical tumor and genetic markers—including CEA, CA-125, CA-19-9, PSA, AFP, LDH, BRCA1, BRCA2, RB1, MEN1, and MEN2A—emerging approaches also consider epigenetic modifications, such as DNA methylation and mRNA expression, as predictors of malignant transformation. Moreover, biomarkers linked to depression, including cytokines, hormones, oxidative stress mediators, and neuropeptides, are gaining attention for their potential relevance in both diagnosis and prevention [40].

To provide a clearer overview, the tumor, genetic, and inflammatory biomarkers integrated into the platform are summarized in Table 1.

### 2.2. Inflammatory Markers and Oncogenic Pathways

Chronic inflammation disrupts homeostasis and plays a central role in the pathogenesis of diverse conditions, including atherosclerosis, cardiovascular disease, osteoporosis, rheumatoid arthritis, and certain malignancies [41,42]. Middle-aged and elderly individuals often exhibit elevated circulating levels of pro-inflammatory cytokines, notably interleukin-6 (IL-6) and tumor necrosis factor-α (TNF-α), together with increased C-reactive protein (CRP), an acute-phase protein synthesized by the liver in response to IL-6 [43,44].

Prospective studies suggest that chronic stress accelerates biological aging by perpetuating systemic low-grade inflammation [45,46]. The rising prevalence of cancer with age can be attributed not only to cumulative genetic and environmental insults but also to the physiological decline of the immune system, further exacerbated by psychosocial stressors and reduced adaptive capacity. This phenomenon of immune-senescence affects both innate and adaptive immunity. The thymus, which produces naïve T lymphocytes, undergoes progressive involution after the age of 50 and is largely ineffective by the age of 60 [47,48,49].

As a result, older adults display a reduced pool of naïve T cells for responding to novel antigens, while memory T cells become predominant [50,51,52]. Although natural killer (NK) cell numbers may increase as a compensatory response, their cytotoxic capacity declines, contributing to the higher incidence of infections and cancer observed in elderly populations [53,54,55]. NK cell senescence has been associated with chronic inflammation, immunosuppressive cytokines, and persistent antigenic stimulation by tumor cells [56].

Furthermore, under conditions of chronic stress, pro-inflammatory genes in leukocytes are upregulated, leading to sustained cytokine release that promotes both tumor initiation and metastatic progression [57,58]. In this context, inflammation emerges as a pivotal oncogenic mechanism, with biomarkers such as IL-6, TNF-α, C-reactive protein (CRP), erythrocyte sedimentation rate (ESR), and fibrinogen representing clinically relevant indicators for integrative cancer risk assessment.

### 2.3. Psychological Profiling and Stress Vulnerability

#### 2.3.1. Conceptualization of Stress in Oncogenesis

The evaluation of psychological vulnerability profiles is an important component in understanding cancer susceptibility. Relevant factors include stress levels, perceived stress, state and trait anxiety, depression, and the presence of Type C personality traits. The association between stress, depression, and cancer, as well as the role of personality in pathophysiological mechanisms, has been widely recognized. Individual resistance and adaptation to stress largely depend on cognitive appraisal and interpretation of reality, leading to highly variable responses. Consequently, psychological profiling represents a valuable tool in cancer risk assessment.

Chronic stress predisposes individuals to depression and anxiety, both of which are implicated in oncogenesis. This relationship has been extensively documented in interdisciplinary fields such as Psycho-Neuro-Immunology and Psycho-Neuro-Endocrinology [59,60]. Stress, defined by Hans Selye as a “general adaptation syndrome” and by Lazarus and Folkman as “cognitive and behavioral efforts to reduce, master or tolerate external or internal demands,” can trigger anxiety, fatigue, frustration, and sadness, producing both somatic and psychological repercussions [61,62].

At the biological level, chronic stress activates the hypothalamic–pituitary–adrenal (HPA) axis through the limbic system, generating immunosuppressive and pro-inflammatory effects [63,64]. Corticotropin-releasing factor (CRF) stimulates the pituitary gland to release adrenocorticotropic hormone (ACTH), which in turn induces the adrenal cortex to synthesize corticosteroids [65]. Stress hormones contribute to DNA damage, increase p53 degradation, and promote carcinogenesis. In parallel, chronic stress-induced inflammation elevates pro-inflammatory cytokines, which further promote tumorigenesis by damaging DNA, impairing DNA repair enzymes, and generating reactive oxygen species (ROS) [66,67,68,69,70,71].

Sustained stress increases the likelihood of cancer cell dissemination and metastasis, partly through selective suppression of Th1 lymphocytes, cytotoxic T cell (CTL)-mediated immunity, and interferon production [72,73,74]. Stressful life events, including bereavement or natural disasters, generate significant physiological consequences, depending largely on individual cognitive and emotional responses [75,76,77,78,79]. Persistent exposure to negative experiences elevates the allostatic load, and while short-term adaptation may occur, prolonged stress eventually induces structural remodeling in the hippocampus, amygdala, and prefrontal cortex, leading to long-term behavioral and physiological changes [80,81].

#### 2.3.2. Interrelationship Between Stress, Depression, and Cancer

Each year, approximately one million new cancer cases are diagnosed among individuals aged 20–39, with stress identified as a contributing factor to this rising incidence [82]. This trend parallels an increase in biological vulnerability across generations. Intergenerational exposure to stress, malnutrition, and childhood trauma has been shown to induce epigenetic modifications in genes regulating the HPA axis and serotonin pathways, thereby increasing susceptibility to multiple physical and psychological disorders [83].

The prevalence of depression has risen significantly among children and adolescents, especially those aged 15–29, in the last two decades. Contributing factors include academic pressure, reduced self-esteem driven by social comparison, particularly in online environments, and artificially induced identity crises. In low- and middle-income countries, where cancer prevalence is already elevated, depression is further aggravated by socioeconomic constraints such as limited access to education, substandard housing, and insufficient social support [84,85].

Depression is a heterogeneous mood disorder with a variable duration and severity, ranging from weeks to years. It is associated with psychological distress, functional impairment, and a reduced capacity for daily activities. Depression may present as a primary mental illness or as part of a dysthymic personality structure and is frequently comorbid with severe illnesses, including cancer. Clinical manifestations include anhedonia, pessimistic thoughts, low vitality, irritability, and heightened reactivity to minor frustrations. Physical signs include a sad or vacant facial expression, persistent frowning, and reduced facial mobility. Cognitive impairments such as diminished self-esteem, self-blame, bradypsychia, bradylalia, hypomnesia, hypoprosexia, and hypobulia further complicate the condition, with visible impact on speech, posture, and movement [86,87].

Evidence increasingly highlights depression as a significant risk factor for cancer. Dysregulation of the HPA axis, impaired immune surveillance, and an altered endocrine function facilitate metastasis and reduce the tolerance to oncological treatments [88,89,90]. Notably, chronic depression combined with a lack of social support has been associated with a decreased cytotoxic activity of natural killer (NK) cells in breast cancer patients, suggesting an impaired immune response that may accelerate disease progression [91,92,93].

#### 2.3.3. Psychological Profiling as a Tool for Risk Assessment

Psychological configurations vary substantially among individuals, leading to differences in perception, coping mechanisms, and behavioral responses to stress. The interaction between stress, depression, anxiety, and cancer underscores the importance of subjective stress appraisal and adaptive capacity.

Protective or “immunogenic” traits—such as self-efficacy, robustness, sense of coherence, self-esteem, internal locus of control, optimism, and religious belief—enhance resilience and may prevent the activation of biomolecular pathways that impair immune and endocrine functions. Conversely, “dis-immunogenic” traits, including anxiety, depression, and neuroticism, predispose individuals to immune dysregulation and heightened disease vulnerability.

Self-efficacy, defined by Bandura in 1982 as the belief in one’s capacity to mobilize cognitive and motivational resources to achieve goals, is strongly associated with resilience and stress resistance [94]. Individuals with high self-efficacy manage adversity effectively, while those with low self-efficacy experience heightened distress and prolonged recovery [95].

The sense of coherence, optimism, and robustness (hardiness) further contribute to psychological resilience. Optimism, described by Carver and Scheier (2010), is associated with an improved prognosis in serious illness, while pessimism correlates with adverse physiological outcomes [96]. Robustness, conceptualized by Kobasa in 1979, encompasses engagement, control, and challenge and is linked to lower susceptibility to stress-related illness [97].

By contrast, a Type C personality, characterized by emotional suppression, passivity, and alexithymia, has been implicated in breast cancer and other malignancies [98,99]. Features such as emotional rigidity, diminished creativity, and loss of humor promote the somatization of distress. Persistent suppression of negative affect may lead to immune dysregulation, including reduced activity of T-helper and NK cells, thereby increasing oncogenic susceptibility [100,101].

Given these associations, comprehensive psychometric evaluation is recommended. Key instruments include the following:

- Perceived Stress Scale (PSS)—assessment of perceived stress.
- Hospital Anxiety and Depression Scale (HADS)—screening for anxiety and depression.
- Montgomery–Åsberg Depression Rating Scale (MADRS)—severity of depression.
- Freiburg Personality Inventory (Type C)—evaluation of Type C traits.
- State–Trait Anxiety Inventory (STAI 1 and 2)—situational and dispositional anxiety.
- Holmes and Rahe Stress Scale—quantification of stress based on major life events.
- Self-Efficacy Scale—evaluation of perceived self-efficacy.

The psychometric and behavioral instruments proposed for integration into the platform are summarized in Table 2.

#### 2.3.4. Facial Micro-Expression Analysis in Depression Detection

##### Clinical Analysis of Facial Expressions in Depressed Patients

Clinical assessment of facial expressions in depression reveals characteristic alterations in regions such as the eyes, gaze, eyebrows, and mouth. Spontaneous micro-expressions, lasting less than 0.5 s, are particularly challenging to detect because they are involuntary [102,103,104].

Depressed individuals frequently present with a downward gaze during rest and speech, drooping eyelids, reduced blinking, and limited cheek elevation when smiling [102,103,104,105]. The lips often angle downward, with corners positioned 5–10° below the neutral line, giving them a thin, inactive appearance with restricted mobility during speech [104,106,107,108]. Smiles typically lack eye involvement, reflecting the absence of the Duchenne smile [109]. Eyebrows are commonly furrowed at rest, with the inner corners drawn together and vertical glabellar wrinkles persisting [103,105,110,111]. Overall, facial movements tend to be slow, rigid, and symmetrical, accompanied by a fixed or glassy gaze and delayed or weak eye contact [112,113].

These clinical features correlate with specific muscle activations described in Paul Ekman’s Facial Action Coding System (FACS), where Action Units (AUs) represent discrete muscular contractions [105,107]. In depression, AU 6, engaging the orbicularis oculi in the Duchenne smile, is typically inactive, producing a forced and flat smile [105,109]. Furrowed brows (AU 1 + 4) and vertical glabellar wrinkles (AU 4) persist at rest, while downward-turned mouth corners (AU 15) and pressed lips (AU 24) reflect sadness and emotional suppression [103,104,106,108,110,111]. In summary, patients with depression exhibit attenuated positive micro-expressions, diminished variation in facial movements, and a predominantly downward or evasive gaze [112,113].

##### Pathophysiological Mechanisms Affecting Facial Expressions in Depression

Altered facial expressivity in depression reflects dysfunction within cortico-striato-thalamo-cortical circuits. The prefrontal cortex—modulated by the amygdala and hippocampus—integrates sensory input from the mediodorsal thalamic nuclei with dopaminergic signals from the ventral tegmental area (VTA), which regulates behavioral motivation [114,115]. The striatum (caudate and putamen) integrates emotional and motor signals, while the globus pallidus and substantia nigra pars reticulata modulate the intensity of motor output [116].

Dopaminergic hypofunction and excessive activity of the indirect basal ganglia pathway produce excessive inhibition of movement, manifesting clinically as hypomimia, reduced smiling, and diminished overall facial expressivity [117,118]. Reduced serotonergic output from the dorsal raphe nucleus further disrupts connectivity among the prefrontal cortex, amygdala, hippocampus, and striatum, contributing to flat affect, diminished facial reactivity, and depressive mood. The thalamus relays these altered signals to motor and premotor cortices, leading to slowed, attenuated facial expressions characteristic of depression [115,119].

#### 2.3.5. Voice Characteristics as Psychological Biomarkers of Depression

##### Clinical Analysis of Speech Patterns in Depressed Patients

Clinical evaluation of speech in depression consistently reveals distinctive alterations, including a flat voice devoid of resonance or emotional expressivity, reduced timbre, and diminished volume. Hidalgo Julia et al. [108] identified both vocal and facial biomarkers of depression, noting a significantly lower mean fundamental frequency compared with non-depressed individuals. Little et al. [120] reported a markedly reduced speech rate, averaging approximately 80 words per minute, while Hidalgo Julia et al. [108] documented minimal tonal variation (flat prosody) and low volume, often bordering on inaudibility. Sanchez et al. [121] highlighted impaired recognition of prosody, and Mundt et al. [122] demonstrated correlations between acoustic features and both depression severity and treatment response. Cummins et al. [123] observed tremulous, hesitant speech patterns with long pauses and delayed responses of up to three seconds. Similarly, Shen et al. [124] described variations in intensity and frequency (jitter and shimmer) as markers of emotional instability in depressed speech.

##### Pathophysiological Mechanisms Affecting Voice and Speech in Depression

The speech alterations observed in depression are mediated by neurobiological mechanisms overlapping with those affecting facial expression. Dopaminergic signaling plays a central role in initiating and coordinating smooth motor execution, including laryngeal and articulatory muscle activity; dopaminergic deficits result in slowed, monotonous speech with prolonged pauses [87].

Serotonergic projections from the dorsal raphe nucleus, together with prefrontal and limbic circuits, regulate affective tone; diminished serotonergic activity produces vocal flatness, reduced intonation, and a “colorless” voice [125]. Norepinephrine release from the locus coeruleus modulates arousal and vocal intensity, and hypofunction results in weak, low-volume, and energy-depleted speech [125].

Dysregulation of inhibitory (GABAergic) and excitatory (glutamatergic) transmission within fronto-striatal circuits further slows motor output, impairing articulation and prosody [126]. In addition, pro-inflammatory cytokines activated through the hypothalamic–pituitary–adrenal axis reduce tryptophan availability for serotonin synthesis and interfere with dopaminergic pathways, leading to delayed verbal responses, frequent pauses, and diminished vocal strength [127].

Collectively, these pathophysiological alterations explain the slowed, monotonous, and emotionally blunted speech that characterizes depression.

#### 2.3.6. Gait and Posture Analysis in Psychological Assessment

##### Clinical Analysis of Gait and Posture in Depressed Patients

Clinical observation of gait and posture in depression consistently reveals characteristic psychomotor alterations. Walking is often slowed (bradykinesia), with a reduced step length, diminished arm swing, and decreased energetic propulsion, reflecting global motor slowing (hypokinesia) and rigidity [86,128,129]. The depressive gait may appear “sluggish” and unstable, particularly during directional changes [86]. In some cases, it mimics Parkinsonian bradykinesia, with asymmetric or reduced arm movements proportional to symptom severity [129].

Postural changes are equally distinctive, typically characterized by a stooped trunk, lowered head, slouched shoulders, and a closed, withdrawn body stance [86,130]. Reduced postural stability is frequently observed, especially during stance changes or while altering the walking direction. Gait initiation may be hesitant, with insecure steps [131,132], and some patients demonstrate “freezing-like” phenomena similar to those encountered in Parkinsonian syndromes [133].

##### Pathophysiological Mechanisms Affecting Gait and Posture in Depression

The psychomotor disturbances observed in depression are primarily attributed to dysfunction of the cortico-striato-thalamo-cortical circuitry. Dopaminergic hypofunction within the basal ganglia enhances activity of the indirect pathway, leading to excessive motor inhibition expressed clinically as small steps, slowed walking, impaired gait initiation, a reduced arm swing, and a stooped posture [87,134].

Serotonin and norepinephrine also contribute to the regulation of motor and emotional tone through their projections to the prefrontal cortex and brainstem. Deficits in these neurotransmitter systems diminish motor energy and postural coordination, manifesting clinically as a slouched posture, lack of bodily expressivity, and rigidity [135]. In addition, prefrontal cortical dysfunction disrupts the integration of emotional and motor signals, impairing movement planning and automatic postural adjustments, which explains the insecure gait, altered walking rhythm, and difficulty changing direction observed in depression [136].

Neuroinflammatory mechanisms, mediated by chronic cortisol elevation and pro-inflammatory cytokine activity, further impair dopaminergic and serotonergic neurotransmission, exacerbating rigidity and deficits in fine motor control [137]. Importantly, although hypokinesia, bradykinesia, and rigidity resemble Parkinsonian motor features, depression does not involve neurodegenerative pathology [138]. Instead, these gait and postural disturbances reflect functional dopaminergic and serotonergic hypofunction within cortico-striato-thalamo-cortical loops [87].

### 2.4. AI-Assisted Models Used in Cancer Risk Evaluation

This section reviews current AI-assisted models designed for cancer risk evaluation, in order to investigate the theoretical possibilities for developing an integrative multimodal computerized platform, able to support, based on the conclusions of our biological and psychological cancer risk factor review, the future development of efficient, reliable and accessible tools for a holistic cancer risk evaluation, which could be extensively used in health systems, especially by primary assistance practitioners and patients. In such an integrated platform, the AI component should provide a computational framework for generating a latent oncological risk profile by integrating multimodal inputs—biological markers, personalization features, psychological assessments, vocal characteristics, postural dynamics, and facial expression metrics—within a unified AI-based model.

**Figure 1:**
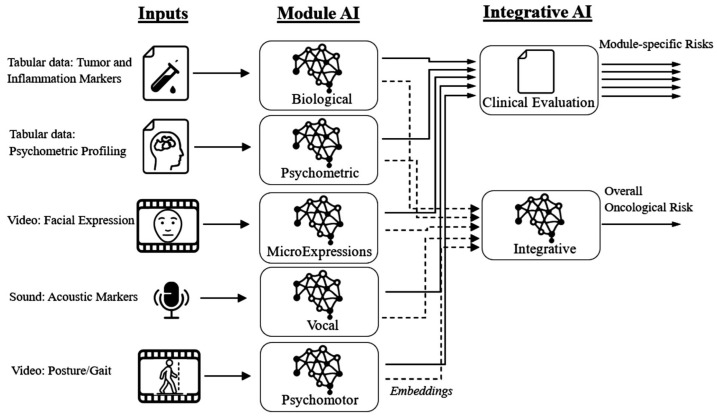
System architecture overview. Legend: The proposed multimodal screening platform integrates five modality-specific encoders: (1) biological module for tumor and inflammation markers, (2) psychometric module for stress, anxiety, and depression profiling, (3) facial expression module for micro-expression analysis, (4) vocal module for acoustic biomarkers, and (5) gait/posture module for psychomotor evaluation. Each encoder generates both an interpretable risk score and a latent embedding. These embeddings are transmitted to the fusion layer, which can operate in late fusion (risk scores combined for interpretability) or early fusion (embeddings combined for richer interactions). The fusion network produces an overall cancer risk score, supported by explainability tools (SHAP values, feature attribution). Data governance includes encryption, access control, and modular deployment via microservices for scalability and security.

A general modular architecture, able to support early detection through interpretable, scalable, and clinically applicable screening, is proposed in Figure 1.

The clear problems encountered by any such type of AI development are inputs’ (management and risk factors) evaluation for scoring, i.e., establishing each factor’s relevance, in itself and contextually, in relation to all other determined factors and with all related clinical and paraclinical existing data. If the data management can be solved, using specific EHRs, such as the NET-DD Convergent Platform (see Section 2.4.8), the problem of an integrated and reliable risk factor evaluation remains to be solved by further research, which can be supported by such a multimodal platform’s future development.

#### 2.4.1. AI Models for Cancer Risk Assessment Using Biological Inputs—Tumor and Inflammation Markers

Recent advances in artificial intelligence have enhanced the integration of tumor and inflammatory biomarkers for cancer risk prediction and prognostic assessment, demonstrating the capacity of AI to capture complex patterns in structured biomarker data for early detection and individualized intervention [139].

A variety of AI approaches have been explored in biomarker-driven prediction. Traditional machine learning models—logistic regression, Random Forest, gradient boosting, and neural networks—have been used to integrate biochemical markers such as γ-glutamyl transferase (GGT) and alanine aminotransferase (ALT) for breast cancer prediction [139]. Multi-cancer screening platforms, such as OneTest, combine tumor-associated proteins (AFP, CEA, CA19-9, CA125, CA15-3, PSA, CYFRA 21-1) with clinical variables and employ models including SVM, k-nearest neighbors, Ridge Logistic Regression, MLP, and J48 decision trees to detect over 20 cancer types [140]. Random Forest and XGBoost have been applied to systemic inflammatory markers and inflammation-related SNPs in colorectal cancer, improving prognostic accuracy [141,142]. Ratios such as neutrophil-to-lymphocyte (NLR) and lymphocyte-to-monocyte (LMR) have been incorporated into Random Forest models for predicting the breast cancer treatment response [143], while population-level studies combining nutritional factors and biomarkers have shown joint influences on cancer susceptibility [103]. Machine learning has also been used to detect persistent post-COVID-19 inflammation using biomarkers such as C-reactive protein and interleukin-6 [144].

Deep learning has likewise been investigated, often alongside other modalities. For instance, Yuan et al. [145] integrated blood-based systemic inflammatory biomarkers—summarized as the systemic immune–inflammatory–nutritional index (SIINI)—with CT radiomics to predict an early response to immune checkpoint inhibitors in non-small-cell lung cancer. Bhattacharya et al. [146] proposed a diffusion-based model combining inflammatory and tumor-related biomarkers with pre-treatment CT scans to forecast immunotherapy outcomes. Collectively, these studies suggest complementary strengths: traditional ML delivers interpretability and actionability on tabular data, whereas deep learning enables richer integration of heterogeneous, multimodal inputs.

#### 2.4.2. AI Models for Cancer Risk Assessment Using Psychometric Inputs—Psychological Profiling and Stress Vulnerability

Psychological factors—including stress, anxiety, depression, and cognitive–emotional traits—are increasingly considered as being possible determinants of the cancer risk, disease trajectories, and outcomes. AI enables the quantitative integration of psychometric data to assess stress vulnerability, emotional resilience, and behavioral patterns relevant to oncology.

Both traditional and deep learning approaches have been applied to psychometric datasets. Logistic regression, Random Forest, gradient boosting, and SVMs have analyzed structured questionnaires (depression/anxiety scales, stress inventories, cognitive-emotional profiles), revealing high-risk psychological configurations associated with adverse outcomes [147,148,149]. Deep learning models (MLPs, RNNs) have been explored, particularly when psychometrics is combined with physiological signals or imaging; for example, Martinez et al. [150] integrated psychometric features with other patient data for earlier breast cancer detection, while Gao et al. [151] used deep learning for comprehensive risk assessment incorporating psychological inputs.

#### 2.4.3. AI Models for Cancer Risk Assessment Using Facial Expression Inputs—Micro-Expressions for Depression Detection

A facial expression module estimates the probabilities of emotional states or behavioral cues (e.g., suppression, distress) from video frames captured during spontaneous speech or stress-inducing tasks. Analysis typically involves three stages: (1) facial landmark detection, identifying key points to capture subtle structural changes; (2) emotion recognition, classifying expressions into discrete or continuous emotional states; and (3) temporal analysis, assessing emotional stability, suppression, and micro-expression dynamics over time. This allows the detection of nuanced psychological signals often imperceptible to human observers.

Convolutional Neural Networks (CNNs) have been foundational for facial expression analysis, with models and datasets including VGG-Face [152], FER+ [153], AffectNet [154], and OpenFace [155]. CNNs excel at static image-based emotion classification. More recently, Vision Transformer (ViT) architectures leverage self-attention to capture long-range dependencies across image patches [156] and can be extended temporally using Video Vision Transformers (ViViTs) [157] to model emotion dynamics across sequences of frames.

Several studies have applied AI specifically for micro-expression-based depression detection. SFTNet, for example, employs a two-stream network to capture both single-frame and temporal micro-expression cues, demonstrating the value of modeling subtle facial dynamics [158]. Region-of-interest-based approaches have been used to detect concealed depression by analyzing localized facial landmarks [159], while AU-based frameworks combining CNNs and LSTMs have been applied to temporal micro-expression recognition [160]. RNN-based models such as FacialPulse further capture temporal sequences of facial landmarks to detect depressive states [161], and detailed analysis of facial action unit sequences has been shown to achieve high accuracy in identifying depression-related cues [162]. Collectively, these studies highlight the effectiveness of AI in identifying subtle, clinically relevant facial expressions associated with psychological distress.

#### 2.4.4. AI Models for Cancer Risk Assessment Using Vocal Input—Acoustic Markers for Depression Detection

A vocal module estimates a psychophysiological score reflecting affective or stress-related states via speech analysis. Inputs are short recordings collected in controlled or semi-naturalistic conditions. Extracted features span prosody (pitch, intensity, rate, pauses), spectral descriptors (formants, MFCCs, spectral flux, harmonics-to-noise), and voice quality (jitter, shimmer, breathiness, roughness), which relate to affective load and stress responses.

A number of AI models have been developed for generic audio classification, which provide foundational architectures for subsequent depression detection. CNN-based architectures such as VGG-ish [163] and YAMNet [164] learn to extract spectral and temporal audio features from raw waveforms or spectrograms. Transformer-based architectures, including wav2vec 2.0 [165], HuBERT [166], and Speech-Transformer [167], leverage self-attention to model long-range dependencies in audio signals, enabling robust pretraining on large-scale speech corpora.

Several studies have specifically applied AI to depression detection using acoustic features. RNN- and LSTM-based models have been employed to capture temporal dependencies in speech from depressed individuals [162,168]. Hybrid CNN-RNN architectures have been used to analyze both spectral and prosodic features for depressive state classification [169]. Additionally, attention-based or Transformer models fine-tuned on depression-specific datasets have shown improved sensitivity to subtle vocal markers indicative of emotional distress.

#### 2.4.5. Gait and Posture Input—Movement Patterns for Psychomotor Evaluation

A gait/posture module quantifies psychomotor function via a probability-based score reflecting deviations in movement patterns (stress-related rigidity, psychomotor retardation, postural instability).

Two data acquisition approaches are commonly employed, depending on the level of detail desired. The first approach relies on short video clips of participants walking, recorded using standard cameras or smartphones. Pose estimation frameworks such as OpenPose [170] for 2D human key points or MediaPipe Pose [171] for 2.5D keypoints are used to extract skeletal representations from these videos. For higher-fidelity analysis, depth-sensing devices such as the Microsoft Kinect [172] or the OrbbecPersee [173] can provide 3D skeletal data, capturing subtle postural sway and millimeter-scale deviations. While depth cameras offer higher precision, they typically require controlled clinical settings and specialized handling.

For automated digital screening platforms, 2D pose estimation is generally sufficient. It allows participants to upload video data independently while still providing meaningful psychomotor metrics. Depth-sensing modules can be integrated as optional enhancements, providing higher fidelity when available, without disrupting the baseline workflow.

Once skeletal keypoints are extracted, they are passed to AI models that analyze psychomotor features, such as gait symmetry, movement smoothness, postural rigidity, and psychomotor retardation. Existing research demonstrates the utility of various AI architectures for this purpose. MLP and Random Forest models have been used for gait analysis in Parkinson’s disease [174], while CNNs, RNNs, and LSTM variants have successfully detected psychomotor retardation and depression from video-recorded movements [175,176]. More recent deep learning approaches include bi-directional LSTMs for fall detection [177], Spatial–Temporal Transformers for gait recognition [178], ViViT for comprehensive psychomotor behavior analysis [179], and cross-attention Transformers for emotion recognition from movement sequences [180]. Several of these models, particularly those for depression detection, explicitly exploit temporal dynamics in movement, highlighting the importance of sequential modeling in psychomotor assessment.

#### 2.4.6. Principles for Modelling an Integrative AI Multimodal Framework

Let us summarizie the above-analyzed AI models, in correlation with the conclusions that, presently, only biological markers have a confirmed relevance in cancer risk evaluation and early diagnostics, as well as in treatment evaluation and evolution monitoring, and that computerized applications, systems, and platforms, currently used worldwide in health both professionally and privately, lack a common informational standardization. Accordingly, we can conclude that an efficient and reliable integrative AI multimodal framework can be modeled only in integration with a standardized computerized platform for integrated health informational management, based on a highly structured, comprehensive, and scalable EHR, containing all the risk factor determinations that are taken into account in analyses—biological and psychological, when adequately acquired as inputs (tabular, images, and vocal recordings).

Also, in order to support the required research on the relevance of psychological determinations in oncogenesis and, consequently, their value in cancer risk evaluation, the AI component of this multimodal platform needs to use biological determinations as references for further validation of psychological factors’ relevance.

**Figure 2:**
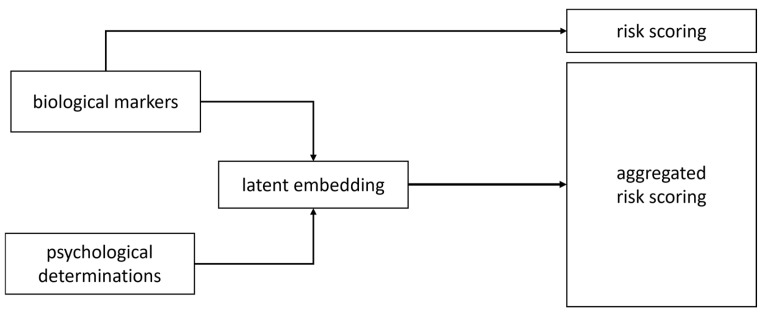
Certification mechanism for psychological determination’s relevance in cancer risk evaluation using biological determinants as references.

In a theoretical multimodal platform design, a biomarker module can process blood-based tumor and inflammatory markers to yield (i) a direct risk estimate and (ii) a latent embedding for downstream fusion. Inputs comprise structured measurements from Section 2.1 and Section 2.2, with continuous features normalized and categorical variables encoded as learnable embeddings. A multilayer perceptron (MLP) encoder trained via back-propagation [181]—augmented with residual connections, layer normalization, and dropout [147]—maps inputs to a compact latent representation capturing nonlinear interactions. The two output heads are as follows: a sigmoid-activated linear layer for a direct risk score, and the latent embedding from the penultimate layer for fusion. This design combines interpretability (a clinical score) with a representation suitable for multimodal integration, while retaining efficiency and modularity for tabular biomarker data (Figure 2).

An MLP has the greatest efficiency and suitability for tabular biomarker data. Unlike classical linear models, the MLP captures complex, nonlinear relationships among biomarkers, enhancing predictive power without the computational and architectural overhead associated with more complex (i.e., full transformer-based) architectures. At the same time, it remains interpretable and modular, allowing straightforward integration into a broader multimodal framework. This separation of biomarker encoding from multimodal fusion ensures the system can be easily updated with additional biomarkers, facilitates interpretability at both the single-modality and fusion levels, and provides clinically actionable insights while maintaining scalability and efficiency.

A psychometric module can process validated instruments (Section 2.3) to generate, primarily, (i) a latent embedding for fusion and, secondarily, (ii) a direct psychometric risk score. Inputs include validated instruments capturing stress, anxiety, depression, resilience, and cognitive–emotional traits. Continuous items are normalized; categorical or ordinal responses are encoded as learnable embeddings. An MLP encoder with residual connections, layer normalization, and dropout transforms inputs into a compact representation supporting the dual-head output. This design balances interpretability (explicit psychometric risk) with flexibility and scalability for evolving questionnaires and composite indices.

For facial expression inputs, a practical approach could use a custom ViT-based architecture with a two-step training strategy:

1. Pretraining on large-scale image-based datasets such as FER-2013 or AffectNet. Although these datasets consist of single images, pretraining allows the model to learn robust spatial facial representations (e.g., eyes, mouth, eyebrow configurations) and general facial features, providing a strong initialization for downstream tasks.
2. Fine-tuning on high-frame-rate micro-expression datasets such as CASME II [182] or SAMM [183], as well as a curated clinical oncology dataset. During this step, temporal sequences are used to capture subtle facial motions and micro-expression dynamics relevant to stress, depression, or oncological risk. This enables the model to specialize in clinically meaningful patterns while mitigating noise from general-purpose datasets.

The architecture can be extended temporally as a ViViT, processing sequences of frames to model micro-expression dynamics over time. The module yields (i) a depression-related risk score and (ii) a facial embedding from the penultimate layer for multimodal fusion—thereby combining general spatial features with clinically relevant temporal cues for comprehensive risk modeling.

For vocal inputs’ interpretation, a practical approach could use wav2vec 2.0 due to its ability to learn rich acoustic representations that can serve as latent embeddings for downstream integration. Pretraining on large-scale general speech datasets provides a robust initialization for capturing fine-grained spectral and prosodic patterns. To specialize the model for oncological-relevant affective and stress-related cues, we propose fine-tuning on a curated clinical dataset, ideally containing speech samples labeled for stress, depression, or psychological distress. It should be pointed out that while wav2vec 2.0 is highly expressive and suitable for multimodal integration, it is also computationally heavier than classical CNN-RNN models and may require careful fine-tuning to avoid overfitting on small clinical datasets. Consequently, alternative approaches could include hybrid CNN-RNN architectures or HuBERT-based models, which can offer a similar performance with potentially lower computational requirements. Additionally, combining pretrained Transformer embeddings with hand-crafted acoustic features (prosodic, spectral, or voice quality) can further enhance the sensitivity to depression-related vocal markers. Outputs would include (i) a direct stress/depression-related risk score and (ii) a vocal embedding from the penultimate layer for fusion.

Finally, for gait/posture analysis, 2D pose estimation followed by a neural feature extractor suffices for remote screening, with optional depth-based modules when available (including on-sensor processing, as in OrbbecPersee [173]. The modular design supports scaling from basic 2D analysis to high-precision 3D assessments without altering the overall workflow, and it yields both interpretable psychomotor scores and latent embeddings for fusion.

To provide a comparative overview of AI techniques across modalities, Table 3 summarizes the primary models that can be employed for biomarker integration, psychometric profiling, facial micro-expression analysis, vocal feature extraction, and gait/posture recognition.

In order to fully operationalize the AI component of the multimodal platform design, an integrative module is required, which combines outputs from modality-specific models—biomarkers, psychometrics, facial expressions, vocal features, and gait/posture—into a unified cancer risk score reflecting latent oncological vulnerability across physiological and psychosocial domains.

Functionally, multimodal fusion can be performed at different stages of the pipeline, depending on whether raw embeddings or final predictions are used. Early fusion involves concatenating or otherwise combining the latent embeddings from each modality model, obtained from the penultimate layer before the modality-specific output. These embeddings retain rich, high-dimensional representations of the modality-specific features, capturing nonlinear interactions within each domain. The fused representation is then passed to a central model—specifically, we propose a Transformer-based architecture—that will learn cross-modal relationships and complex dependencies. It is important to note that the final probability scores produced by each modality are not used for early fusion; these scores are reserved for clinical interpretation and for late fusion applications.

Late fusion, in contrast, operates on the final outputs of each modality, i.e., the probability scores. These scores are combined to produce an overall risk estimate. A variety of AI architectures are suitable for this stage, ranging from lightweight multilayer perceptron to ensemble learning models (e.g., gradient boosting, Random Forests) and more sophisticated Transformer-based architectures capable of modeling inter-modality dependencies. Late fusion benefits from interpretability, as each modality’s contribution to the final prediction can be individually examined and validated.

For initial clinical deployment, late fusion is often preferred for modular validation and transparency. Each modality can be benchmarked against clinical standards, and the overall score can be decomposed to support clinical reasoning. As data scale and validation mature, early fusion may be explored to capture complementary cross-modal interactions potentially missed by late fusion, albeit with increased data and training demands. This staged strategy balances performance, interpretability, and clinical trust.

#### 2.4.7. Limitations of an Insulated AI Multimodal Platform Model

Several limitations should be acknowledged. Risk scores generated by the platform are not diagnostic substitutes but screening tools requiring clinical correlation. Multimodal data acquisition, while comprehensive, remains resource-intensive and may limit accessibility in low-resource settings. Algorithmic bias is a concern if training datasets are imbalanced, potentially leading to disparities in prediction accuracy across populations. Ethical and privacy considerations are central, particularly regarding the use of facial, vocal, and psychometric data. Ensuring informed consent, anonymization, secure data handling, and fairness in algorithmic decision-making is essential. In order to overcome most of these limitations, the possibility of integration with the health informational management convergent NET-DD platform, including the practical development of its theoretical ‘structured informational management concept’, has also been reviewed.

#### 2.4.8. The Convergent Computerized Platforms Approach

Having in mind all AI-assisted limitations and risks, both those presented above and those related to text processing algorithms (Natural Language Processing—NLP), which are of uttermost importance in health data management, as well as the current taxonomic inconsistencies and errors in the overall medical and medical-related data management (such as medical, pharmaceutical, and biotechnological classifications, as well as societal organizational and personal ones), a new, original approach is provided by the application of a ‘systems’ convergence concept [180] in the computerized informational management of our study, using the operational infrastructure of the proprietary NET-DD platform [184,185].

This innovative approach solves, at least, two significant problems: (1) the extensive deployment of our platform’s integrated applications, through convergent virtual networks, interconnecting professionals and patients, both localized and distributed (telemedical application), and (2) the high level of imprecision of all AI-assisted applications in use—with a maximum accuracy of 80% for simple queries, and much less for complex ones—by providing a certified training environment for any type of expert system, machine learning algorithm, and deep learning application control mechanism, exclusively using convergently structured information (as further explained). Merging AI-type developments with ‘computerized structured informational management’ results in the original theoretical concept of ‘structured AI’ applications.

The structured AI concept postulates that any systematic knowledge—in the case here, related to cancer—can be convergently transformed (by applying the convergent informational standard) into unique informational volumes (data and metadata), and then can be aggregated with all the rest of the existing convergent informational volumes, all computerized by a convergent, web-operated platform. Once convergently transformed and aggregated, the entire disposable informational volume can be limitlessly reaggregated and presented for computerized processing. Any such processing generates results, directly in the format of unique informational volumes, maintaining, as such, the convergence of the entirety of preexisting informational volume, while expanding it, progressively and contiguously.

If we state that the entire existing informational volume within a convergent platform has the value of truth, scientifically proven, then all results of its computerized processing have the same value, provided the processing formula or algorithm is correct. Since several computerized processing methods can be used on the same informational volume, it is consequential that results’ variability indicates, with certainty, flaws in one or all applied methods.

This leads to the conclusion that, in theory, an accurate input of convergent, structured informational volumes into any type of computerized processing method applied on them leads to an accurate results evaluation tool, since variant results absolutely indicate methodic inconsistencies.

It is more than clear that the structured AI training concept is initially slow and progressive, but it is also, in compensation, scientifically valuable, safe, and natively efficient in its perfection process, due to the informational dynamic optimization mechanism. Also, most important, it ensures a maximum-efficiency computerized data processing of all aggregated informational volumes, for a limitless amount of processing formulas and algorithms (AI type included). Actually, the research process for developing operational machine learning and convergent hybrid applications, along with convergent expert systems for assisted medical and pharmaceutical decision, is ongoing, with results being expected shortly.

#### Convergent Transformation of Informational Management for AI-Assisted Multimodal Cancer Screening Platform—The Integrated Specific EHR

The convergent systemic transformation states that any system, natural (physical) or non-physical—such as ideas, concepts, beliefs, and theories—must be fully informationally described, structurally and functionally, in order to have a reasonable meaning and human utility—individual and social. So are the health system and all its components, including diseases like cancer. All scientifical and practical informational management of health systems lacks integrability due to one essential fact: taxonomical inconsistency and error, meaning there are multiple identifications, classifications, and codifications for the same thing, as well as significant classification and codification flaws, or various forms of gravity. The direct result is the impossibility to efficiently process the information gathered within and regarding the health system, anywhere in the world—even more so for computerized processing. This state of facts hinders the efficient development of all types of information technology systems used or useful to health systems’ management and health research.

In order to solve this problem in an organic manner, the systems’ convergent concept postulated that all systems can be transformed into uniquely informationally defined ones, by applying a set of principles, based on (1) using a uniform informational standard for all systems, namely the ‘convergent informational standard’, and (2) determining the commonality of all other elements of a system, namely the ‘convergence element’.

The convergent informational standard cannot by applied abruptly on all existing systems, as they are defined and informationally managed to date, but can be progressively and compliantly implemented, using the method of ‘dynamic informational optimization’—so clear acceptance and utilitarian problems induced by an abrupt implementation attempt are avoided.

In sum, the convergence concept defines any system as a number of components (or elements), stably bound together—determining the systemic structure—and stably functioning in relation to themselves—internal function—and in interaction with other external systems—external function—in a cause–effect relation. The system, its components (that can be indivisible, as ‘objects’ or a composite, and hence other systems), and its external interactions (that can be only systemically defined as well) are all completely informationally described, proportionally to humans’ existing overall knowledge—something not humanly known cannot be a system. The convergent informational standard applies to all informational descriptions of systems, ensuring the uniqueness of their cognitive senses—the meanings human intelligence (objectively and subjectively, even pathologically) attributes to them.

Practically, the convergent informational standard defines any type of information as an ‘informational volume’, which must have a unique identification—the denomination of a system and of its structural and functional components—and a stable parametrization of its structure and function, each parameter being uniquely identified and precisely measurable, both in quantitative and qualitative terms, directly measured or, if not possible, indirectly—by comparison with directly measurable parameters.

The indivisible type of parameter is defined as a ‘data’, described by identification, which is unique in all existing systems, and by its measurement values, expressed in numbers and related measurement units.

Complex parametrization is carried out by combining a set of unique data, resulting in a ‘data package’, also uniquely identified for all systems.

Systemic description is performed by one or more uniquely identified combinations of data, data packs, and metadata, always fully ‘commented’—each metadata must be associated within a uniquely identified data pack, which fully informationally describes its content and significance.

Back to information’s convergent definition, for any system, the general convergent transformation can be mathematically expressed:

ⱯS = IVs V Vf

where IVs (structural informational volume) and IVf (internal-functional informational volume) can be any combination of data, or data packs, with or without associated metadata.

The same goes for general systemic function:

IVi → S → IVo

where IVi is the input interactional informational volume, and IVo is the output structural and functional-interactional informational volume.

It is important to stress here that, in the case of any system ‘S’, the output informational volume defines the systemic stability in a measurable manner, i.e., the system ‘S’ is stable only if

IVo⊃S

Also, it is of the highest relevance that there are numerous systems we can convergently transform about which we know nothing more than their identification and external function—the case of the black box, where

S = S and IVi → S → IVo

Hence, results the generality of convergent informational management, including the integrated and the computerized ones, regardless of the degree of systemic informational parametrization, which is proportional to the amount of knowledge we possess about the given system.

By applying the convergent transformation to any modern health system, first, we determine that the ‘convergence element’ is the ‘medical act’, informationally described as all direct interactions between a patient and one or more practitioners, starting with the unique identification of the two parts systemically involved, the first being further described as a biological system, the latter as an object, part of one component of the integrated health system.

Results show that the medical act is, informationally, a succession of professional–patient systemic interactions, described by specific convergent informational volumes, aggregated in the EHR. All informational volumes common to all medical acts constitute the standard EHR, while all other informational volumes, as is the case of our multimodal platform, aggregate further into one specific EHR.

**Figure 3:**
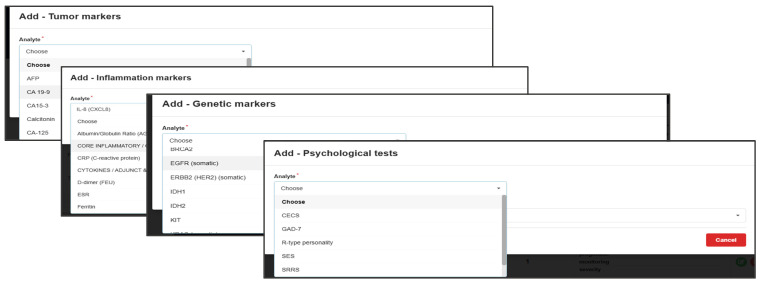
Recording flow of data.

**Figure 4:**
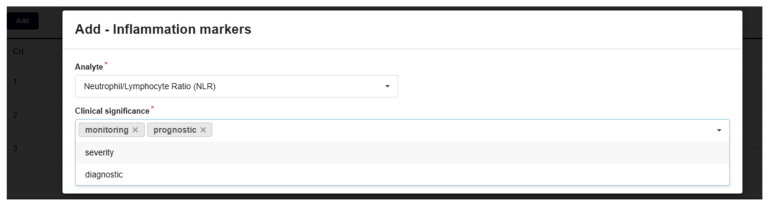
Clinical signification convergent record using standard optimizable selection lists.

**Figure 5:**
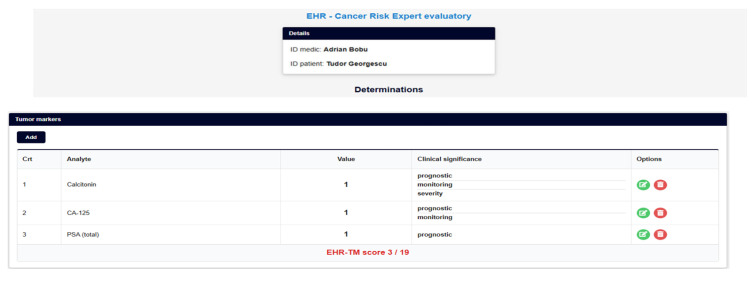
Standard EHR structures’ preview.

**Figure 6:**
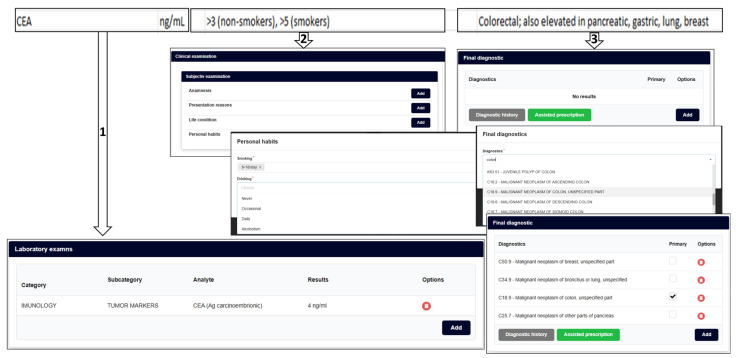
Convergent transformation of unstructured information—example for CEA tumor marker integrated into specific EHR, for a structured AI database. Each unstructured informational volume related to transformation is structured using the convergent HER standard, according to its sense. So, the CEA tumor marker unstructured format (upper) is transformed into three structured informational volumes, which are to be further processed within any computerized platform, using any type of formula and algorithm, especially the AI -type, for optimized results (analytical and predictive). In the structuring process, all generally accepted data management standards are integrated—in this particular case, the IDC-10 International Codification of Disease for potential diagnostics of the CEA tumor marker positive determination (4 ng.ml being relevant in a non-smoker patient case).

So, the information presented in Figure 1 and Figure 2 is convergently transformed (Figure 3, Figure 4, Figure 5 and Figure 6), in order to become the infrastructure for further AI structured developments—seeing all previously presented methods and algorithms, using a specifically configured ‘EHR for cancer-risk expert evaluation’.

### 2.5. Illustrative Clinical Scenario (Theoretical Example)

From a clinician’s perspective, a patient may present with apparently modest biochemical changes (e.g., slightly elevated IL-6 and borderline CRP), together with subtle psychometric alterations (mild depression score) and minimal gait/posture changes that are not clinically striking at the bedside. In routine clinical practice, such a case would not be flagged as “probable cancer”. However, the structured multimodal AI model described in this manuscript would not analyze each marker in isolation, but would weight and fuse them into a unified representation. This would allow the system to detect a “weak but convergent” vulnerability pattern earlier than a conventional human assessment, thus enabling earlier monitoring and referral for confirmatory testing.

Convergent transformation is shown in Figure 2 of all four risk-evaluation determinations—tumor markers (TM), genetic markers (GM), inflammatory markers (IM), and psychological tests (PT).

Convergent parametrization is also applied, for all determinants—related to any specific medical act by the patients’ and professionals’ unique identification, using only three criteria on this particular operational model: (1) determination of positive result, (2) pathological relevance—selecting only malignancies (Figure 4), and (3) evolutionary perspective of selected pathology (Figure 5).

For scientifical rigor, given the conclusions of this review, the simplest mathematical model has been applied—scoring of positive relevant determinations.

Schematically, the convergent transformation applied on an unstructured training database for a multimodal AI platform model for cancer risk evaluation, resulting in a structured AI model, is presented in Figure 6.

It outlines the observation that structured informational management, integrated into any type of AI application, increases accuracy while eliminating the need for NLP use.

#### 2.5.1. A Mathematical Simple Expert Convergent Model for Further AI-Assisted Multimodal Cancer Screening Platforms

The specific EHR we have configured allows for standard computerized processing in order to mathematically express certifiable indicators for cancer-risk determination, as well as to consistently demonstrate all further potentially successful research developments.

Based on a simple scoring (as implemented in the convergent platform), two conclusions result: (1) simple scoring (Table 4) has only limited relevance, resulting in as many risk indexes as determinant categories (in our case, four), since ‘total score’ values cannot be relevant by simple summation (so X,Y1 → n are, in fact, impossible to calculate), and (2) in order to increase the relevance of risk indexes, two methods can be applied, by combining each determinant and each analyte with its own specific relevance (Table 5).

The first method relies on a two-factor formula: RI(A)(%) = A f(RA1, RA2), and the second one is multifactorial RI(D) = D f(RD, RA1 → n), respectively, RI(T) = f(RD1 → n)—where RI = risk indicator, A = analyte, D = determination category, T = total, f = computerized data processing, indifferent to the calculation type (mathematical formula, computational algorithm, AI model), and X,Y1 → 4 = unknown values, depending on fx,y(A1 → n, D1 → n).

So, in order to obtain optimal informational computerized processing results, besides the specific convergent taxonomy determination and subsequent convergent transformation, enhanced precision and specificity must be added.

Precision is enhanced by determining one or more clinical relevance (R) scale, to each determinant and analyte, in relation to their clinical significance, pathological (malignancy or other), evolutive (predictive, monitoring, severity, etc.), or other. More detailed (parametrized) clinical significance improves the accuracy of risk-index calculated results.

Specificity is enhanced by adding to each pathology category (such as malignancy, which we selected for this application) all other related ones, for each determination and analyte, and also by classifying each pathology to the specific disease level (what malignancy exactly it is—parotid or colon cancer—and what type—type of carcinoma or type of sarcoma).

Both precision and specificity are enhanced by integrating all information contained in the standard HER with the particular one provisioned for the cancer-risk evaluation one, with valuable cross-relations being available as such.

**Figure 7:**
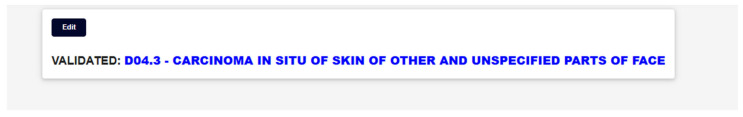
Example of validated risk-evaluation results, mapped with ICD-10 codification.

This type of processing not only provides valuable practical information, by conventionally using the convergent platform as part of the multimodal one, but also orients future AI-integrated developments and aids in their accuracy evaluation. For this specific AI-oriented purpose, we integrated into the EHR the pathology validation function, compatible with any classification for pathology—such as the selected ICD-10 one (Figure 7).

If validation versus invalidation is acquired, for all specific EHRs for cancer-risk expert evaluation, the maximum accuracy can be obtained for any type of use, either as an expert system or an AI predictive one. In fact, this completes the practical demonstration of our structured AI concept, which we aim to fully develop in the near future. It certifies that the structured AI model determines the contiguous and dynamically optimizing enhancement of any AI-type application training, and hence of accuracy, as opposed to current algorithms, which determine a sequential training progress, with a high variability of accuracy improvement.

#### 2.5.2. Clarification Note (Methodological Position Statement)

This manuscript is conceptual and does not report the implementation or empirical validation of a multimodal platform. The psychological determinants discussed (stress, anxiety, depression) are considered potential modulators of vulnerability, not proven causal drivers of oncogenesis. Facial, vocal, and gait-derived signals are therefore interpreted here strictly as indirect proxies of human systemic status, not as direct oncological biomarkers. Consequently, this review does not claim that multimodal fusion outperforms established biomarkers; future empirical studies are needed to quantify the true additive predictive value of hybrid models.

## 3. Conclusions and Future Directions

Computational methods are already integrating part of health and health systems’ function, as the widely used term of ‘bioinformatics’ proves. The drawbacks of current health programs, but also of general health management, are partially the consequence of limitations of the computational methods used to administrate them, informationally or in their absence (mostly as a primary assistance to patient relations). They are also the direct consequence of conceptual flaws, accessibility to healthcare providers and patients, as well as lack of social adherence.

Using the example of cancer risk evaluation, in our review, we conclude that extending our attention to accessible, computerized, and more integrative methods has great potential, if properly conducted, in scientifical and technological terms.

In the particular case of integrating psychological evaluation in a cancer risk assessment protocol, a combined computerized platform, operated as a Platform as a Service (PaaS) and integrating an AI multimodal component, could not only provide an accessible tool for professionals and patients but also support the research required to perfect itself scientifically, certifying the objective role of psychological factors in oncogenesis, and hence the value of psychological determinations in cancer risk evaluation and also in cancer management.

Convergent platforms, as much as any conventional health data management systems, provided they use acceptable informational standards and informational structuring, provide accessibility for a multimodal computerized approach to cancer risk evaluation, while current AI developments in the field of psychological factors’ evaluation, related to cancer risk and oncogenesis, are already mature for developing an operational AI component, according to the principles for a theoretical design we presented.

Of course, the clinical adoption of an AI-assisted multimodal cancer screening platform requires rigorous validation across multiple datasets and clinical settings. Performance should be assessed using discrimination metrics (area under the ROC curve, precision–recall curve, F1-score), calibration metrics (Hosmer–Lemeshow test, calibration plots, Brier score), decision-curve analysis to quantify the clinical net benefit, and both cross-validation and external validation to ensure generalizability. Ablation studies, in which biomarkers, psychometric data, or behavioral signals are selectively removed, will help determine the incremental contribution of each modality to the overall prediction.

From the clinical significance standpoint, such a platform would introduce a paradigm shift in cancer-risk assessment by uniting biological, psychological, and behavioral dimensions within a single digital framework. Its clinical significance can be summarized as follows:

- Early risk detection: Identifying latent vulnerabilities before clinical manifestation increases opportunities for preventive intervention.
- Personalized screening: Risk profiles integrating biomarkers and psychosocial indicators support precision medicine strategies.
- Interdisciplinary integration: By bridging oncology, psychology, and biomedical engineering, the platform fosters a holistic view of cancer prevention.
- Non-invasive digital design: Data can be collected remotely using smartphones, webcams, or simple blood tests, facilitating telemedicine and reducing patient burden.
- Longitudinal monitoring: Regular follow-up enables the dynamic tracking of patient trajectories, linking early detection to proactive management.

To advance this platform from theory to practice, several priorities should be addressed:

1. Dataset creation and curation: Large-scale, multimodal, clinically annotated datasets are essential for training and validating robust AI models.
2. Longitudinal studies: Prospective cohorts integrating biological and psychosocial data will clarify causal relationships between stress, depression, and oncogenesis.
3. Explainability and interpretability: Transparent AI models, employing methods such as SHAP values or counterfactual reasoning, are required for clinician trust and regulatory approval.
4. Cross-cultural validation: Since psychological and behavioral markers vary across cultures, generalizability must be tested in diverse populations.
5. Clinical workflow integration: Implementation science approaches will be needed to align the platform with healthcare infrastructure, policy, and reimbursement models.

For real-world adoption, the proposed platform requires a translational pathway bridging research and clinical practice. Initial implementation could be piloted within oncology departments of academic hospitals, where both biomarker testing and psychological expertise are available. Integration with electronic health records (EHRs) would allow seamless data transfer, while patient interfaces could be deployed through secure mobile or web applications for remote assessments. Multidisciplinary clinical teams—including oncologists, psychologists, biomedical engineers, and data scientists—should collaborate to establish workflows for data acquisition, interpretation, and follow-up.

At a broader level, partnerships with mobile health technology providers and telemedicine networks could enable population-level deployment, expanding the access to preventive screening in underserved regions. Reimbursement frameworks, pilot programs supported by national health agencies, and outcome-based validation studies will be essential for sustainable clinical integration.

### 3.1. Ethical and AI Bias Considerations

The integration of AI into cancer screening raises critical ethical and regulatory challenges. One of the foremost concerns is algorithmic bias, which may arise from imbalanced training datasets that underrepresent certain demographic or cultural groups, potentially leading to disparities in screening accuracy. To mitigate this risk, diverse and representative datasets must be used, alongside fairness-aware machine learning techniques and continuous post-deployment monitoring.

Privacy and informed consent are equally central. Sensitive data—such as facial video, vocal recordings, and psychometric responses—must be anonymized or pseudonymized, encrypted during transmission and storage, and accessed only under strict governance protocols. Patients should be clearly informed about how their data are used, stored, and analyzed, and they must retain the right to withdraw at any time. Furthermore, the explainability and transparency of AI models are vital for clinician trust. Techniques such as SHAP values, feature attribution, and counterfactual explanations should be embedded in the system to allow physicians and patients to understand the rationale behind risk predictions. Addressing these ethical dimensions not only ensures compliance with regulations such as GDPR or HIPAA but also supports equitable and trustworthy deployment of the platform in diverse healthcare settings.

### 3.2. Future Outlook

The convergence of AI and computerized structured informational management with oncology and behavioral sciences promises to open up new perspectives in preventive oncology. With expanding datasets and more refined models, multimodal AI platforms may evolve into decision-support systems for oncologists, psychologists, and primary care providers. In the near future, pilot studies could be conducted in university hospital oncology departments and, most of all, in primary assistance healthcare providers, leveraging both clinical and psychological expertise. Collaborations with technology companies could enable population-level deployment through mobile applications, while interdisciplinary research grants in behavioral oncology and medical AI may accelerate progress. Ultimately, such platforms have the potential to transform cancer prevention from a reactive to a proactive process, especially by stimulating the direct, extensive, and cohesive involvement of patients.

As a next step, a feasible operational direction is a small pilot feasibility study focused on a limited subset of multimodal determinants (e.g., IL-6, CRP, PHQ-9 score, vocal tone variability), to test whether stable signal fusion is technically achievable and quantifiable. Such preliminary work would provide the empirical foundation required before scaling to broader multimodal clinical validation.

## 4. Final Remarks

The proposed integrative platform model we designed demonstrates there are ways to develop accessible integrated computerized systems able to combine tumor and inflammatory biomarkers with psychometric assessments and behavioral indicators—including facial micro-expressions, voice analysis, and gait/posture evaluation—to provide a holistic perspective on cancer vulnerability.

Its convergent architecture allows for continuous updating as new biomarkers and data modalities emerge, ensuring adaptability over time. It also illustrates a clear path toward personalized, accessible, and ethically responsible analytical and, further, prospective cancer risk assessment.

With proper validation and interdisciplinary collaboration, the long-term vision is to reduce the global cancer burden through proactive, patient-centered prevention strategies, as well as preventive, screening, and early detection protocols, with the ultimate goal of shifting cancer prevention into a proactive, patient-centered paradigm that redefines standards in global oncology.

Through adding the structural AI developments to the multimodality of the platform, promising directions are foreseen for enhancing predictive accuracy, for scientifically certifying the etiological–pathogenic, diagnostic, and predictive values of psychological determinations in oncology, and for efficiently integrating with currently used e-health applications and systems.

## Figures and Tables

**Table 1:** Summary of biomarkers integrated into the platform.

Category	Examples	Clinical Relevance	References
Tumor markers	CEA, CA-125, CA-19-9, PSA, AFP, LDH	Early detection, monitoring tumor burden	[33,34,35,36,37,38,39]
Genetic markers	BRCA1, BRCA2, RB1, MEN1, MEN2A	Hereditary cancer risk	[38]
Inflammatory markers	IL-6, TNF-α, CRP, ESR, fibrinogen	Chronic inflammation, cancer progression	[41,42,43,44,45,46]

**Table 2:** Psychological and behavioral indicators.

Domain	Key Features	Tools/Methods	Clinical Relevance	References
Stress and anxiety	Perceived stress, state/trait anxiety	PSS, STAI	Links to immunosuppression and oncogenesis	[59,60,61,62,63,64]
Depression	Mood, facial expressivity, voice	HADS, MADRS, digital voice/facial analysis	Predictor of cancer outcomes	[82,83,84,85,86,87,88,89,90,91,92,93]
Personality traits	Type C, low self-efficacy, low coherence	Freiburg Inventory, Self-Efficacy Scale	Modulate immune function, stress response	[94,95,96,97,98,99,100,101]

**Table 3:** AI methods applied in different modalities.

Modality	Model(s)	Data Type	References
Biomarkers	MLP, Random Forest, XGBoost	Structured tabular	[139,140,141,142,143,144,145,146]
Psychometrics	Logistic Regression, MLP, RNN	Questionnaire data	[147,148,149,150,151]
Facial Analysis	CNN, ViT, ViViT	Image/video	[152,153,154,155,156,157,158,159,160,161,162]
Voice	wav2vec2.0, CNN-RNN hybrids	Audio	[162,163,164,165,166,167,168,169]
Gait/Posture	OpenPose, ViViT, LSTM	Video skeletal data	[170,171,172,173,174,175,176,177,178,179,180]

**Table 4:** EHR record example for simple scoring.

	TM	GM	IM	PT	Total Score	Risk Index (%)
Patient 1: TG	3/19	5/24	3/17	4/6	15/66	X1
Patient 2: AB	6/19	6/24	6/17	5/6	23/66	X2
Patient 3: SN	4/19	4/24	4/17	6/6	18/66	X3
Patient 4: CE	5/19	4/24	5/17	3/6	17/66	X4

Legend: TM = tumor markers; GM = genetic markers; IM = inflammatory markers; PT = psychological tests; Xi = variable values per patient; TG, AB, SN, CE = patient ID.

**Table 5:** Comparison of results obtained via methods for enhancing results’ specificity.

	TM		GM		IM		PT		Total Score		Risk Index (%)
	Score	Relevance	Score	Relevance	Score	Relevance	Score	Relevance	Score	Relevance	
TG	3/19	R1.1	5/24	R1.2	3/17	R1.3	4/6	R1.4	15/66	R1	Y1
AB	6/19	R2.1	6/24	R2.2	6/17	R2.3	5/6	R2.4	23/66	R2	Y2
SN	4/19	R3.1	4/24	R3.2	4/17	R3.3	6/6	R3.4	18/66	R3	Y3
CE	5/19	R4.1	4/24	R4.2	5/17	R4.3	3/6	R4.4	17/66	R4	Y4

Legend: TM = tumor markers; GM = genetic markers; IM = inflammatory markers; PT = psychological tests; Ri = variable relevance per determination; Yi = variable values per patient; TG, AB, SN, CE = patient ID.

## Data Availability

The original contributions presented in this study are included in the article. Further inquiries can be directed to the corresponding authors.

## Abbreviations

The following abbreviations are used in this manuscript:

ACTH	Adrenocorticotropic Hormone
AFP	Alpha-Fetoprotein
AI	Artificial Intelligence
AUC	Area Under the Curve
AU	Action Unit (Facial Action Coding System)
BRCA1/2	Breast Cancer Gene 1/2
CEA	Carcinoembryonic Antigen
CA-15-3/CA-19-9/CA-125	Cancer Antigens 15-3, 19-9, 125
CD	Cluster of Differentiation
CNN	Convolutional Neural Network
CRF	Corticotropin-Releasing Factor
CRP	C-Reactive Protein
CTL	Cytotoxic T Lymphocyte
DL	Deep Learning
DNA	Deoxyribonucleic Acid
EHR	Electronic Health Record
ESR	Erythrocyte Sedimentation Rate
FACS	Facial Action Coding System
FER	Facial Emotion Recognition (Dataset)
GGT	Gamma-Glutamyl Transferase
GM	Genetic Marker
HADS	Hospital Anxiety and Depression Scale
HIPAA	Health Insurance Portability and Accountability Act
HPA Axis	Hypothalamic–Pituitary–Adrenal Axis
HR	Hazard Ratio
HuBERT	Hidden-Unit BERT (Speech Model)
ICD-10	International Classification of Diseases, 10th Revision
IL-6	Interleukin-6
IM	Inflammatory Marker
KNN	k-Nearest Neighbors
LDH	Lactate Dehydrogenase
LMR	Lymphocyte-to-Monocyte Ratio
LSTM	Long Short-Term Memory Network
MADRS	Montgomery–Åsberg Depression Rating Scale
MEN1/MEN2A	Multiple Endocrine Neoplasia Type 1/Type 2A
MFCC	Mel-Frequency Cepstral Coefficients
ML	Machine Learning
MLP	Multilayer Perceptron
MRI	Magnetic Resonance Imaging
NLP	Natural Language Processing
NK Cells	Natural Killer Cells
NLR	Neutrophil-to-Lymphocyte Ratio
PSS	Perceived Stress Scale
PT	Psychological Test
PSA	Prostate-Specific Antigen
RF	Random Forest
RNA	Ribonucleic Acid
ROC	Receiver Operating Characteristic
ROS	Reactive Oxygen Species
RNN	Recurrent Neural Network
SVM	Support Vector Machine
STAI	State–Trait Anxiety Inventory
SIINI	Systemic Immune–Inflammatory–Nutritional Index
SN	Substantia Nigra
TNF-α	Tumor Necrosis Factor Alpha
TAA	Tumor-Associated Antigen
TSA	Tumor-Specific Antigen
TM	Tumor Marker
ViT	Vision Transformer
ViViT	Video Vision Transformer
VTA	Ventral Tegmental Area
WHO	World Health Organization
wav2vec 2.0	Self-supervised Speech Representation Model
XGBoost	Extreme Gradient Boosting
